# Forty years of changes in scientific publishing: from conflict of interest to generative AI

**DOI:** 10.1093/haschl/qxag125

**Published:** 2026-05-21

**Authors:** Howard Bauchner

**Affiliations:** Department of Pediatrics, Boston University Chobanian and Avedisian School of Medicine, Boston, MA 02118, United States; National University of Singapore,Singapore

**Keywords:** communication, scientific publishing, data-sharing, preprints, open access

## Abstract

There have been many changes over the past 40 years in scientific publishing, including concerns about conflict of interest, the debut of the Internet, data-sharing, open-access publishing, mandated registration of randomized clinical trials, the challenges of reproducibility and replicability, preprints, and the use of generative AI to help create and review manuscripts. These issues and the future of scientific publishing are discussed.

The last 40 years have brought dramatic changes to scientific publishing, beginning with concerns about conflicts of interest (COIs) in 1984 to the use of generative artificial intelligence (AI) in the 2020s to assist with creating and reviewing manuscripts. This summary is based on my experience as the Editor in Chief (EIC) of *Archives of Disease in Childhood* (ADC) (2004-2011) and the *Journal of the American Medical Association* (JAMA) and JAMA Network (2011-2021).

## 1984

In a *New England Journal of Medicine* (NEJM) editorial in 1984, Arnold Relman, the EIC, stated, “Nevertheless it is obvious that business arrangements have an increasing role in medical research….”^1^ He declared that NEJM was instituting a new policy in which authors were requested to acknowledge all funding sources as well as any other business associations relevant to the research. In 2009, the Institute of Medicine released a report on COI, and in 2017 JAMA devoted an entire issue to the subject.^2,3^ Issues that continue to be debated include the following: if there is no such thing as a potential COI, whether authors should declare all COIs or only those that they believe are relevant, has compensation for consulting arrangements become so substantial that the amounts should be reported, and should journals that produce podcasts ensure that those interviewed declare COIs.^4^

## 1993—World Wide Web/Internet

In 1993 the European Organization for Nuclear Research (CERN) released software that allowed simple access and use of the Internet.^5^ Journals were transformed, changing from print to digital releases. Print circulation declined for most journals, and over the past 3 decades, journals have become communication networks, with electronic releases via the internet worldwide. For example, in 2011 JAMA had approximately 15 000 followers on social media, while in 2025 JAMA had over 1.7 million followers, and reached over 2 900 000 people with its Table of Contents and online first alerts, dwarfing the approximately 300 000 print editions in the 1990s.^6^ In addition, the Internet has allowed journals to reach readers in new formats, including via social media and podcasts.

## 1996—Data-sharing

In 1996 the Bermuda principle was established, ensuring that all gene sequences would be made publicly available.^7^ In anticipation of the end of the Human Genome Project in 2001, leading investigators from around the world wanted to ensure that individual genes would not be patented. The clinical world took note and, in 2018, after urging from various groups, the International Committee of Medical Journal Editors (ICJME) suggested that every randomized clinical trial (RCT) be released with a data-sharing statement. Numerous groups have since mandated data-sharing, and in 2022, the US government, mandated in the coming years (instituted in 2025) that data underpinning National Institutes of Health (NIH)–supported manuscripts, and those of all federal agencies, must be made freely available.^8^ Whether widespread sharing of clinical data advances science and leads to improved patient outcomes is not yet clear.

## 2002—Open access

In 2001, the Open Science Institute, convened a meeting in Budapest, and established the principles of open access (OA), which is a foundational principle of Open Science.^9^ The Budapest Open Access Initiative (BOAI) was published in 2002, stating “the literature that should be freely accessible online is that which scholars give to the world without expectation of payment.” There are now over 22 000 OA journals.^10^ Approximately half of the 3 million manuscripts published in the biomedical literature are now published as OA. One of the challenges has been the proliferation of OA journals that are focused on profit—that is, the more a journal publishes the more income is generated. How to maintain editorial integrity and independence remain a challenge.

## 2005—Mandated registration of RCTs

In 2005, ICMJE mandated that RCTs needed to be registered prior to publication.^11^ This dramatically transformed the reporting of RCTs, among the most influential article types. In addition to registration, now a protocol and statistical analytic plan (SAP) accompany many RCTs. Registration was necessary because of concerns that authors could choose those outcomes that were found to be “significant” a form of *P*-hacking. Few changes have had such a positive effect on the ethical reporting of research.

## 2012—Reproducibility and replicability

In 2012, Begley and Ellis^12^ stated that laboratory-based studies that underpin cancer research could not be reproduced. This was followed in 2015 by a report that many psychology studies could not be reproduced.^13^ These observations crystallized concerns about both reproducibility, defined by the NIH as “the ability of independent researchers to test a hypothesis through multiple methods and consistently achieve results that confirm or refute it, ensuring findings are generalizable and robust across different approaches” and replicability as “the ability to perform the same experiment or study using the same methods and conditions to achieve the same result.”^14^

Reproducibility and replicability remain a work in progress, in part because of limited funding and scholarly recognition, and the complexity of reproducing clinical studies. With clinical studies, the intent of the additional analyses needs to be clear, to reproduce the findings exactly, which would require the same dataset and knowledge of how variables were coded and analyzed, or to find consistency of results from various datasets.

## 2019—Preprints

In 2019, medRxiv (free online repository) was launched.^15^ Although preprints existed in many disciplines for years—for example, arXiv was launched in 1991, primarily for math and physics, medRxiv signaled that clinical medicine, despite some early resistance by investigators and editors also had to engage with preprints. The use of medRxiv increased dramatically during the COVID-19 pandemic. Although many tout the benefit of preprints, such as faster access to scientific information, as evident by various publications during the COVID-19 pandemic, early descriptions of the clinical effectiveness of hydrochloride and ivermectin, later proved to be ineffective, were posted on preprint servers. Preprints have been associated with an increase in the number of citations of the published manuscript. Assessing the risks and benefits of preprint servers during a pandemic is not representative of how servers function during other times.

## 2020s

Beginning sometime in the 2020s investigators began to use generative AI to assist in scientific discovery and the creation of manuscripts. This has been associated with an increase in manuscripts from publicly available, large datasets. For example, among 9 publicly available datasets there has been an estimated 11 577 excess publications based on established trends.^16^

Generative AI is now used to produce manuscripts, and increasingly to review manuscripts. For example, Perlis et al^17^ reported that, over a 27-month period, declared use of AI in 105 538 manuscripts submitted to the JAMA Network increased from 1.71% to 5.97%. In a recent survey, 53% of 909 scientists acknowledged using large language models when asked to review manuscripts, despite instructions from some journals that these models should not be used.^18^ In the future, investigators will increasingly use AI to assist in producing manuscripts, and journals will use AI to assist in reviewing them.^19^ AI has the potential to detect undeclared use of AI by authors, assess compliance with hundreds of reporting guidelines, detect fabricated references, and help editors deal with other challenges.

## The future of scientific publication

Open-access publication in its various forms will continue to increase. Indeed, the NIH mandate that investigators must now post their accepted manuscript in PubMed Central (PMC) will likely be followed by similar policies of funders worldwide, if they have not already done so. This will create a problem of potentially 3 versions of a manuscript—a preprint, an accepted manuscript, and a published manuscript. In addition, posting an accepted manuscript in PMC, which is a repository, is very different than a published manuscript being freely available—OA—on a journal website.

Preprints are here to stay. Most journals now accept that authors may post results on a server prior to submission. Journals should insist that authors detail any major differences between the preprint and publication.

AI will increasingly be used to generate manuscripts and review manuscripts. Human peer review, which is known to be inefficient, biased, and often ineffective in detecting scientific misconduct, is no longer feasible given the number of submitted manuscripts.

Reproducibility and replicability will continue to be a challenge. It should be possible to reproduce a laboratory-based experiment, but finding consistent results across clinical studies is more complicated. Given the massive amount of data, it may be possible to find any outcome an individual prefers.

Fewer clinicians will read the primary medical literature. Rather, they will turn to AI for summaries of the literature. For example, NEJM and JAMA now share their content with Open Evidence, which is currently free to physicians.

Funders and journals will mandate registration of cohort studies whose intent is to define a causal relationship, such as trial emulation studies. As demonstrated by the benefits of trial registration and the massive amounts of data now available for analysis, registering certain types of observation studies is increasingly important.

Change is inevitable—and often good. It is difficult, if not impossible, to determine if the changes noted above have improved science and benefitted patients, but some have (Bermuda principle, trial registration) and others are more debatable (preprint servers, OA publishing).

## Supplementary Material

qxag125_Supplementary_Data

## Supplementary material

There is no supplementary material is available at *Health Affairs Scholar*.
